# Epidemiology and survivorship of soft tissue sarcomas in adults: a national cancer database report^a^

**DOI:** 10.1002/cam4.288

**Published:** 2014-07-08

**Authors:** Robert M Corey, Katrina Swett, William G Ward

**Affiliations:** 1 Guthrie ClinicSayre, Pennsylvania; 2 Division of Public Health Sciences, Wake Forest Baptist HealthWinston Salem, North Carolina

**Keywords:** American College of Surgeons, epidemiology, national cancer database, sarcoma, soft tissue, survival

## Abstract

The National Cancer Data Base (NCDB) of the American College of Surgeons gather demographic and survival data on ∼70% of cancers in the USA. We wanted to investigate the demographic and survivorship data on this potentially more representative cohort of patients with soft tissue sarcomas. We selected 34 of the most commonly encountered soft tissue sarcomas reported to the NCDB, provided that each entity contained a minimum of 50 cases. This report summarizes the demographic and survivorship data on 63,714 patients with these 34 histologically distinct soft tissue sarcomas reported to the NCDB from 1998 to 2010. The overall survivorships of these sarcomas were near the lower limits of many prior reports due to the all-inclusive, minimally biased inclusion criteria. The overall best prognosis was Dermatofibrosarcoma NOS (not otherwise specified). (5-year survivorship 92%). The worst prognosis was Dedifferentiated Chondrosarcoma (5-year survivorship 19%). New observations included Biphasic Synovial Sarcoma demonstrating a better 5-year survivorship (65%) compared to spindle-cell synovial sarcoma (56%, *P* < 0.031) and Synovial Sarcoma, NOS (52%, *P* < 0.001). The demographic and 2- and 5-year survivorship data for all 34 soft tissue sarcomas are presented herein. This extent of demographic and survival data in soft tissue sarcomas is unprecedented. Because of the large number of cases and the inclusive nature of the NCDB, without restriction to certain stages, categories, or treatments, it is less subject to selection bias. Therefore, these data are thought to be more reflective of the true overall prognosis given the current management of sarcoma across the NCDB contributing sites.

## Introduction

Much of what we know and teach about the epidemiology of musculoskeletal sarcomas derives from retrospective series collected at large teaching hospitals. Many of these series have been reported in peer-reviewed literature and have been included in published textbooks 1. The single largest report of which we are aware included 26,758 soft tissue sarcomas 2, however, this series did not report the survivorship of patients diagnosed with these sarcomas.

Because of the relative infrequency of musculoskeletal sarcomas, few institutions will gather sufficient numbers to provide thorough epidemiologic and descriptive data. Further advances regarding soft tissue sarcoma epidemiology, especially rare types of soft tissue sarcomas, will come from concerted efforts to collect data within tumor registries. The two largest tumor registries in the United States are the National Cancer Data Base (NCDB) of the American College of Surgeons (ACOS) and the National Cancer Institute's Surveillance, Epidemiology and End Results (SEER) program.

The NCDB is a joint project of the American Cancer Society and the Commission on Cancer of the ACOS. The ACOS has executed a Business Associate Agreement that includes a data-use agreement with each of its Commission on Cancer-accredited hospitals. The NCDB, established in 1989, is a nationwide, facility based, comprehensive clinical surveillance resource oncology data set that currently captures ∼70% of all newly diagnosed malignancies in the US annually http://www.facs.org/cancer/ncdb/index.html.

We wanted to determine from the NCDB the demographic and survivorship information for patients with 34 soft tissue sarcomas. We specifically wanted to report these data for these histologic subtypes of sarcomas as adequate numbers of patients with many of these infrequently encountered sarcomas do not exist within single institution-based series. For instance, controversy exists within the literature as to whether biphasic synovial sarcoma has better, worse, or similar prognosis to Nonbiphasic Synovial Sarcoma 3–6. Only through analysis of an extensive database of cases from a less-biased source can such an accurate assessment of the potential prognostic differences be performed.

We hypothesized that these data would confirm previously established demographic characteristics of patients with these soft tissue sarcomas but that in the least common sarcomas, demographic distribution may differ from prior reports. We also hypothesized that the data would represent more accurate prognostic data as there would likely be less selection bias than that present in individual center-based reports. We believe, as others have suggested http://skepdic.com/posoutbias.html, that researchers and centers tend to favor reporting good results, and since surgical series typically only include reported cases subjected to attempted surgical cures, we hypothesized that the survivorship rates would be lower than rates frequently reported in individual institution-based series.

## Methods

The NCDB currently collects data for all cancer patients diagnosed or given first course treatment at facilities accredited by the Commission on Cancer. Each year, there are ∼1500 Commission on Cancer-accredited cancer centers in the United States. This number varies slightly from year to year. (http://www.facs.org/cancer/ncdb/index.html) The NCDB has never reported the collected data on soft tissue sarcomas, despite the extensive accumulation of demographic and survivorship data on such sarcomas.

Before 1997, submission of cancer patient records to the NCDB was voluntary and was open to all cancer facilities in the Unites States. Beginning in 1997, data collection was mandated as a requirement of CoC-accredited program, allowing nonaccredited programs to continue to voluntarily report cases to the NCDB. In 2000, data collection was further limited to only CoC- accredited programs. Data quality checks against all cases were conducted at the local level as well as the NCDB on receipt of data submissions. Nationally standardized cancer registry edit reports are generated by the NCDB and returned to participating hospitals for review, correction, and resubmission when data errors or data conflicts were found.

The senior author applied for research access to the NCDB data on bone and soft tissue sarcomas through the NCDB- beta Participant User File (*β*PUF) research program, for the purpose of studying and reporting the available data on musculoskeletal sarcomas.

From 1998 to 2010, 87,087 soft tissue sarcomas were reported from 1588 hospitals to the NCDB, a mean number of 54.8 cases per facility over a 13-year period. We selected 34 of the 239 soft tissue sarcomas reported to the NCDB that are commonly encountered by orthopedic oncologists. We selected only those histologic entities that included a minimum of 50 distinct cases.

These 34 soft tissue sarcomas were extracted from the NCDB using the appropriate second and third editions of the WHO International Classification of Disease for Oncology (ICD-0-2/3) site (C40.0-C40.9, C41.0-C41.9) and histology codes. Data were abstracted using coding guidelines documented in the Registry Operations and Data Standards manual for cases diagnosed before 2003 and the Facility Oncology Registry Data Standards manual for diagnosis year 2003 and beyond.

The 1998–2010 annual reports to the NCDB included 63,714 cases of soft tissue sarcomas with the 34 histologies listed in the data tables. The most common soft tissue sarcoma in our report is malignant fibrous histiocytoma (MFH), which includes 12,754 cases over a 13-year period. MFH is followed by Sarcoma NOS (not otherwise specified) and Myxoid Liposarcoma, which occurred at frequencies of 7842 cases and 3996 cases, respectively.

Data analyzed include patient gender, age (Note: the *β*PUF program only shared data on patients of at least 18 years of age), race, date of initial diagnosis by year, anatomic site of primary tumor, tumor grade (well differentiated, moderately differentiated, poorly differentiated, undifferentiated), tumor size, and 2-year and 5-year survivorship. Anatomic site coding is divided into 19 anatomical sites, including varying locations within the peripheral and autonomic nervous system, connective and soft tissue lesions, and overlapping lesions within multiple anatomic locations (Tables S1–S3).

**Figure 1:**
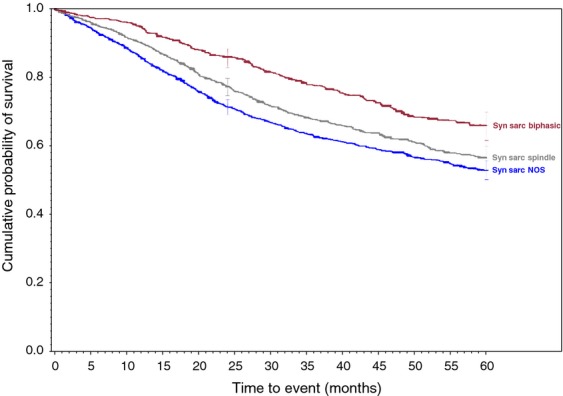
Synovial sarcoma family of soft tissue sarcomas.

**Figure 2:**
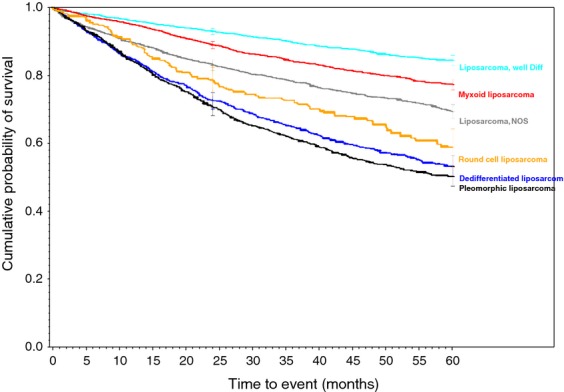
Liposarcoma family of soft tissue sarcomas.

**Figure 3:**
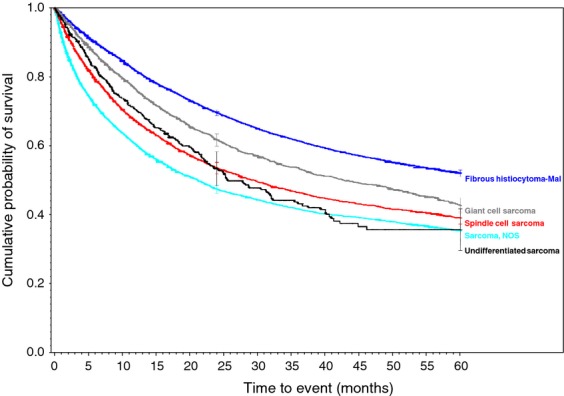
Malignant fibrous histiocytoma (MFH), giant cell, and poorly differentiated soft tissue sarcomas.

**Figure 4:**
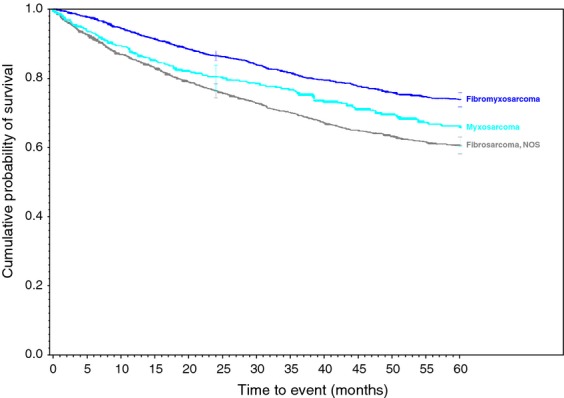
Myxoid and fibrous family of soft tissue sarcomas.

**Figure 5:**
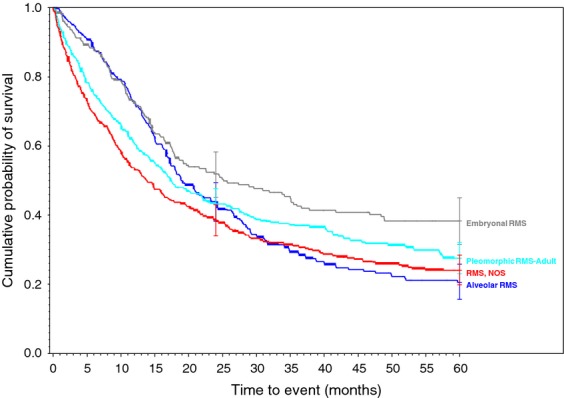
Rhabdomyosarcoma family of soft tissue sarcomas.

**Figure 6:**
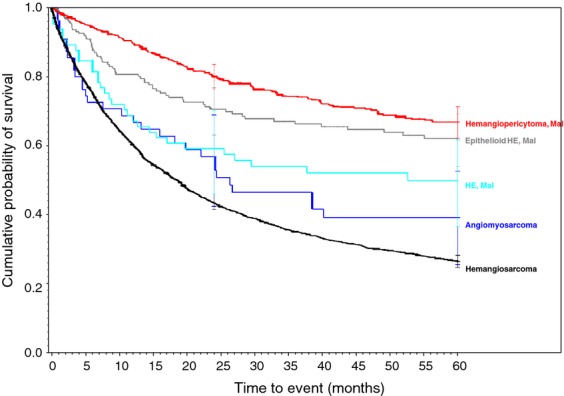
Vascular family of soft tissue sarcomas.

**Figure 7:**
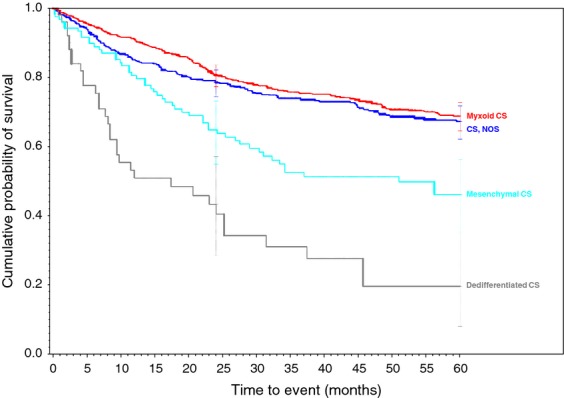
Chondrosarcoma family of soft tissue sarcomas.

**Figure 8:**
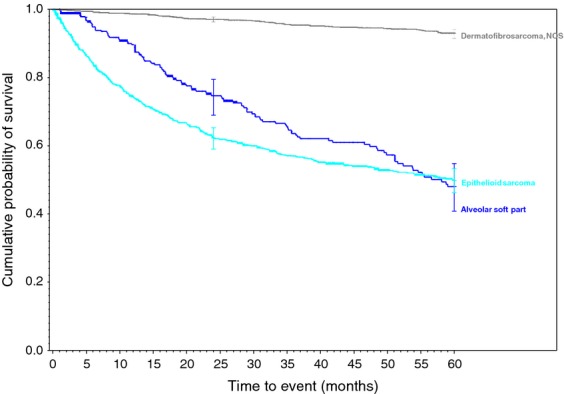
Miscellaneous soft tissue sarcomas.

**Figure 9:**
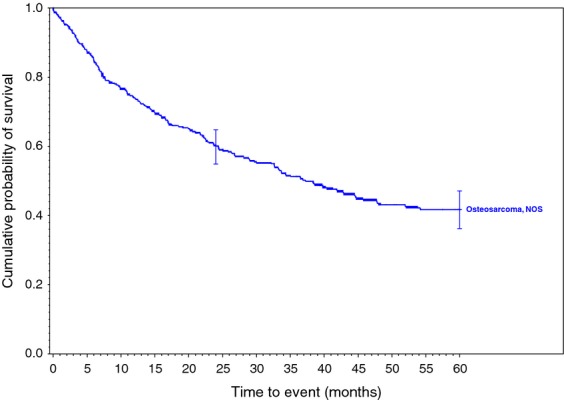
Osteosarcoma, NOS of soft tissue.

**Figure 10:**
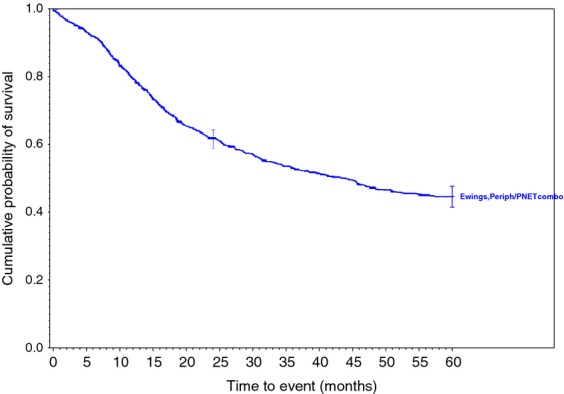
Ewings family of soft tissue sarcomas.

Survival rates were calculated for each of the 34 histologic types of soft tissue sarcoma. Descriptive statistics were generated for all measures, including means, ranges, and standard deviations for continuous measures and frequencies and proportions for categorical data. Overall survival (OS) was calculated from the date of diagnosis to the last known date of follow-up or the date of death. Sarcoma-specific death was not reported. Estimates of survival were calculated by using the Kaplan–Meier (product-limit) method and the log-rank test was used to assess statistical significance. Cox proportional hazards models were fit to assess survival differences adjusting for demographic and clinical covariates. Statistical significance was defined as *P* < 0.05. All analyses were performed using SAS 9.3 (Cary, NC). We grouped certain similar histological subtypes together and combined other histologic sarcomas to create the ten graphs (Figs.1 and 2 with text, Figs. 3–10 available as supplement) illustrating the Kaplan–Meier survivorship curves of the 34 sarcomas.

Data reported to the NCDB are retrospective in nature. No patient or physician identifiers were collected as part of this study. Case identification information (facility identification number and local registry accession number) was collected for administrative purposes only. The ACOS has executed a Business Associate Agreement that includes a data-use agreement with each of its CoC-accredited hospitals. Results reported in our study were in compliance with the privacy requirements of the Health Insurance Portability and Accountability Act of 1996 as reported in the Standards for Privacy of Individually Identifiable Health Information, Final Rule (45 CFR Parts 160 and 164). Although this study is IRB-exempt, the IRB in place at each of the sites employing the authors reviewed the research proposal and confirmed IRB exemption status.

## Results

The demographic data for each sarcoma, the particulars of which is one of the primary questions sought by this study, are presented in Tables 1 and 2 and in Tables S1–S4. Gender, age, and race are displayed in Table 1. The overall gender predilection demonstrated that soft tissue sarcomas are more common in males than in females, by a 1.23 to 1.00 ratio. Two soft tissue sarcomas demonstrated an overall female predilection. Malignant hemangiopericytoma and hemangiosarcoma displayed a sex predilection of 1.00:1.27 and 1.00:1.08 male: female ratio, respectively. Three sarcomas displayed nearly equal sex distribution: myxosarcoma, osteosarcoma NOS, and rhabdomyosarcoma NOS. Excluding the two female predominant tumors and the three gender equal tumors, the male/female ratio for the remaining 29 sarcomas was 1.26 to 1.00. The age distributions are displayed in Tables 1 and 2. (The NCDB *β*PUF program did not share data on patients under 18 years of age.) The tumor demonstrating the oldest median age of all ages in years at time of diagnosis is Angiomyosarcoma (69 median, 68.1 mean); followed by MFH (68 median, 65.9 mean) and Giant-Cell Sarcoma (65 median, 64.2 mean). The two youngest reported patient populations were Alveolar Rhabdomyosarcoma (25 median, 32.5 mean) and Alveolar Soft Part Sarcoma (27 median, 30.9 mean). Table 2 demonstrates that the most common decade of age for adult patients to be diagnosed with soft tissues sarcoma is during the eighth decade of life, followed by seventh and sixth decade of life.

**Table 1:** Demographic and survivorship data for 34 soft tissue sarcomas

Histology	N	Median	Male	Female	White	Black	Hispanic	Asian/PI	Other/Unknown	2-year survival (%)	5-year survival (%)
Alveolar soft part sarcoma	306	27.0	155	151	169	78	34	18	7	74	47
Angiomyosarcoma	55	69.0	27	28	45	4	6	0	0	56	39
Chondrosarcoma, dedifferentiated	52	61.5	28	24	37	5	4	2	4	43	19
Chondrosarcoma, mesenchymal	125	39.0	72	53	86	21	13	3	2	64	46
Chondrosarcoma, myxoid	754	56.0	481	273	573	94	51	19	17	80	68
Chondrosarcoma, NOS	527	58.0	327	200	425	34	30	17	21	78	67
Dermatofibrosarcoma, NOS	2417	43.0	1216	1201	1425	670	162	78	82	97	92
Ewings (including PNET)	1340	32.0	752	588	1040	72	147	55	26	61	44
Fibromyxosarcoma	2835	60.0	1508	1327	2332	221	137	67	78	86	73
Fibrosarcoma, NOS	1977	55.0	1024	953	1480	274	130	42	51	76	60
Fibrous histiocytoma, malignant	12754	68.0	7154	5600	10573	1072	618	286	205	69	52
Giant-cell sarcoma	3415	65.0	1958	1457	2789	310	160	87	69	61	42
Hemangioendothelioma, epithelioid, malignant	191	48.0	88	103	150	18	13	5	5	70	62
Hemangioendothelioma, malignant	66	50.0	37	29	51	9	5	0	1	59	49
Hemangiopericytoma, malignant	529	56.0	238	291	418	47	37	17	10	80	67
Hemangiosarcoma	3372	69.0	1623	1749	2764	319	148	80	61	43	26
Liposarcoma, Dedifferentiated	1509	66.0	971	538	1251	98	93	32	35	72	53
Liposarcoma, myxoid	3996	49.0	2354	1642	2998	381	431	82	104	88	77
Liposarcoma, NOS	2624	64.0	1466	1158	2050	259	197	68	50	83	69
Liposarcoma, Pleomorphic	1551	65.0	902	649	1263	153	73	35	27	70	50
Liposarcoma, round cell	410	49.0	245	165	315	42	31	14	8	78	58
Liposarcoma, well differentiated	3794	63.0	2207	1587	3032	318	240	107	97	92	84
Myxosarcoma	484	61.5	243	241	371	57	32	18	6	80	65
Osteosarcoma, NOS	438	59.0	213	225	323	60	36	8	11	60	41
Rhabdomyosarcoma, alveolar	348	25.0	192	156	229	64	37	13	5	43	20
Rhabdomyosarcoma, embryonal	254	37.0	160	94	181	39	24	5	5	51	38
Rhabdomyosarcoma, NOS	505	52.0	262	243	333	87	64	13	8	38	24
Rhabdomyosarcoma, Pleomorphic, adult type	526	63.0	344	182	414	57	32	12	11	43	27
Sarcoma, epithelioid	1035	46.0	647	388	796	119	74	24	22	62	49
Sarcoma, NOS	7842	63.0	4232	3610	6079	919	479	213	152	47	35
Sarcoma, spindle-cell	3927	63.0	2063	1864	3010	458	261	100	98	53	39
Synovial sarcoma, biphasic	732	40.0	387	345	538	84	66	23	21	85	65
Synovial sarcoma, NOS	1820	41.0	953	867	1316	212	197	49	46	71	52
Synovial sarcoma, spindle cell	1204	41.0	616	588	850	120	171	28	35	77	56
Totals	63714		35145	28569	49706	6775	4233	1620	1380		

**Table 2:** Age distribution of 34 soft tissue sarcomas

	Age at diagnosis							
	
Histology	18–29	30–39	40–49	50–59	60–69	70–79	80+	Total
Alveolar soft part sarcoma	184	67	28	16	5	6	0	306
Angiomyosarcoma	2	3	2	6	15	7	20	55
Chondrosarcoma, Dedifferentiated	0	5	9	10	16	6	6	52
Chondrosarcoma, mesenchymal	34	30	20	15	10	11	5	125
Chondrosarcoma, myxoid	32	84	143	177	163	97	58	754
Chondrosarcoma, NOS	34	66	75	105	96	111	40	527
Dermatofibrosarcoma, NOS	411	588	610	441	184	132	51	2417
Ewings (including PNET)	592	283	219	122	64	43	17	1340
Fibromyxosarcoma	182	262	407	534	545	491	414	2835
Fibrosarcoma, NOS	196	256	336	357	285	336	211	1977
Fibrous histiocytoma, malignant	273	580	1258	2042	2531	3241	2829	12754
Giant-cell sarcoma	82	158	349	661	769	769	627	3415
Hemangioendothelioma, Epithelioid, malignant	20	31	50	37	26	22	5	191
Hemangioendothelioma, malignant	7	12	13	11	8	8	7	66
Hemangiopericytoma, malignant	25	69	96	127	100	79	33	529
Hemangiosarcoma	129	173	291	464	642	879	794	3372
Liposarcoma, dedifferentiated	14	41	154	300	387	359	254	1509
Liposarcoma, myxoid	350	739	1007	779	522	390	209	3996
Liposarcoma, NOS	44	152	350	543	570	602	363	2624
Liposarcoma, pleomorphic	26	68	166	301	364	390	236	1551
Liposarcoma, round cell	27	84	107	79	52	44	17	410
Liposarcoma, well differentiated	49	172	544	826	943	807	453	3794
Myxosarcoma	14	31	81	89	99	86	84	484
Osteosarcoma, NOS	35	35	60	91	88	77	52	438
Rhabdomyosarcoma, alveolar	206	47	40	19	15	14	7	348
Rhabdomyosarcoma, embryonal	96	42	39	28	27	11	11	254
Rhabdomyosarcoma, NOS	126	61	44	75	68	76	55	505
Rhabdomyosarcoma, Pleomorphic, adult type	20	34	64	93	129	105	81	526
Sarcoma, epithelioid	219	178	186	171	110	106	65	1035
Sarcoma, NOS	483	590	982	1369	1404	1615	1399	7842
Sarcoma, spindle cell	237	325	487	684	728	810	656	3927
Synovial sarcoma, biphasic	202	152	165	106	71	27	9	732
Synovial sarcoma, NOS	489	382	400	251	153	87	58	1820
Synovial sarcoma, spindle cell	319	263	260	193	88	61	20	1204
Total	5159	6063	9042	11122	11277	11905	9146	63714

The overall race distribution is displayed in Table 1 and include 78% white, 10% black, 6% Hispanic, 2.5% Asian/Pacific islander. EPub Tables S1–S3 display the primary anatomic sites. The most common anatomic site where these soft tissue sarcomas are diagnosed is in the connective and subcutaneous tissues of the lower limb and hip. The overall distribution of soft tissue sarcomas in this database by year of diagnosis is displayed in ePub Table S4. This shows that the overall numbers of soft tissue sarcomas reported to the NCDB increased over this time span. Overall, the most common defined sarcoma grade at the time diagnosis was coded as poorly differentiated (25%). The grade and size of each sarcoma at the time of diagnosis is displayed in Epub Table S5.

There has been some variation in the number of sarcomas reported to the NCDB over this 13-year period. In order to investigate this interval change, the percent increase or decrease in the reported sarcomas was calculated using an average of the first 3 years of this series (1998–2000) compared to the last 3 years of this series (2008–2010). The average number of cases reported in the first 3 years was 4491, compared to an average of 5362 reported in the last 3 years. By this analytic methodology, the overall number of reported soft tissue sarcoma increased by 19% over this 13-year interval.

The 2-year and 5-year survival for patients with each type tumor is displayed in Table 1. The overall best prognosis is for patients diagnosed with dermatofibrosarcoma NOS (97% 2-year, 92% 5-year) and is followed by Well-differentiated liposarcoma (92%, 84%). The three sarcomas demonstrating the worst prognosis appear to be rhabdomyosarcoma, NOS (38%, 24%), dedifferentiated chondrosarcoma (43%, 19%), and pleomorphic rhabdomyosarcoma, adult type (43%, 27%).

Biphasic synovial sarcoma was determined to demonstrate a better 5-year survivorship (65%) than spindle-cell synovial sarcoma (56%, *P* < 0.031) and Synovial Sarcoma, NOS (52%, *P* < 0.001). A similar progressive decrease in survivorship was demonstrated within histologically-related subtypes of the Liposarcoma family. As more and less well-differentiated subtypes of Liposarcomas were compared, they presented a range of survivorships depending on the histology and its level of differentiation. The survivorships of the well-differentiated subtype, myxoid subtype, round cell subtype, and pleomorphic subtype were compared to liposarcoma NOS and ranged from 84% in the well-differentiated subtype (*P* < 0.010) to 50% in the Pleomorphic subtypes (*P* < 0.630 when compared to the 69% 5-year survivorship of liposarcoma, NOS). Additionally, the 5-year survivorship in the myxoid subtype was 77% (*P* < 0.562) and 58% in round cell liposarcoma (*P* <0.038 when compared to liposarcoma, NOS).

## Discussion

This report represents the single largest series of patients for each of the 34 soft tissue sarcomas. It also contains the most highly populated survivorship curves for patients with these 34 soft tissue sarcomas. While the database has limitations, the demographic and survival data are unprecedented in number and duration. (Our search strategy employed searching the textbook references cited earlier. In addition, the authors performed a PubMed search investigating the largest collections of each of the 34 soft tissue tumors. No larger series for each sarcoma were found.)

This series demonstrated demographic data (age/gender/race) in accordance with most previously published reviews of large case series for each of the entities 1. It confirmed that soft tissue tumors are more common in males than in females (1.23 to 1.00). Excluding the five sarcomas that did not display male predominance, the sex predilection was 1.26 to 1.00. However, Malignant Hemangioendothelioma and Hemangiosarcoma were more common in females, displaying sex predilection of 1.00–1.27 and 1.00–1.08, respectively. The female sex predilection of malignant hemangioendothelioma and hemangiosarcoma is a new finding as previous series have found nearly equal gender distributions 7,8. The overall race distribution is displayed in Table 1 and includes 78% white, 10% black, 6% Hispanic, 2.5% Asian/Pacific islander, generally mirroring that of the population of the United States (78% white, 13% black, 17% Hispanic, 5% Asian/Pacific islander; http://quickfacts.census.gov/qfd/states/00000.html) 9.

The survivorship data includes more patients diagnosed with most of these soft tissue sarcomas than any prior case series. This enables us to present a more accurate representation of patient survivorship than noted in previously reported smaller series of patients, which are often derived primarily from single institution databases and are often limited to patients with sarcomas of certain stage, anatomic location, and/or treatment. The single largest report of which we are aware included 26,758 soft tissue sarcomas 2. It included the demographic information containing 615 Synovial-Cell Sarcomas, 3085 Liposarcomas, and 4577 MFH. It also reported the anatomic location of each soft tissue sarcoma, as well as gender and race distribution. However, it did not report the survivorship of these soft tissue sarcomas. The largest series reporting survivorship data on Synovial-Cell Sarcoma contained 243 patients 10. The current series contains 3755 patients diagnosed with Synovial-Cell Sarcoma. The largest series of Liposarcoma containing survivorship data that we could find contained 155 patients 11, while this series contains 12,367 patients diagnosed with Liposarcoma. The largest series of MFH containing survivorship data contained 338 patients 12, while this series contains 12,367 patients diagnosed with MFH.

Overall, most of our 5-year survivorships are within the range of previously published data. For example, the reported 5-year survival of synovial sarcomas can range from 36% to 76% 10,13–17. Our data demonstrated survivorships of 52%, 56%, and 65% for Synovial Sarcoma, NOS, Spindle Cell, and Biphasic, respectively. However, the reported survival rates tended towards the lower ranges of previously reported rates. Two tumors were found to demonstrate poorer survival than previously reported. The lowest previously reported 5-year survivorship for Dedifferentiated Chondrosarcoma was 24% 18, while these data revealed a 5-year survivorship of 19%. Similarly, the 5-year survivorship of Epithelioid Sarcoma has been reported to be only as low as 50% 19–22, while our data demonstrated a slightly lower 5-year survivorship of 49%.

One tumor was found to demonstrate a higher 5-year survivorship than previously reported. Alveolar Rhabdomyosarcoma survivorship has been quoted as low as 2% ranging to 10% 23,24 while our data revealed a 5-year survivorship of 20%. This may reflect that most reported series are in the pediatric population, whereas this database is limited to patients 18 years of age or older.

As displayed in the ePub online version of this manuscript, Graphs 1–10 display statistically significant survivorship curves for many of the histologic subtypes within different families of soft tissue sarcomas. For example, Graph 2 displays that the survivorship of individuals diagnosed with Liposarcoma is dependent upon the histologic subtype of Liposarcoma. As displayed, the 5-year survivorship can range from 84% (*P* < 0.010) in those patients diagnosed with well-differentiated Liposarcoma to 50% (*P* < 0.630) in those patients diagnosed with pleomorphic Liposarcoma (*P* values derived from survivorship curve comparison to that observed with Liposarcoma, NOS). Similarly, Graph 1 demonstrates that the 5-year survivorship of synovial sarcoma can range from 65% (biphasic synovial sarcoma) to 52% (synovial sarcoma, NOS, *P* < 0.001). These differences in survivorship within the same family of sarcomas provide prognostic information for future patient care**.**

The overall number of reported soft tissue sarcoma increased by 19% over this 13-year interval. This increase in the overall number of soft tissue sarcomas reported over the 13-year time interval may be due to a number of different reasons: including an increase in the actual number of soft tissue sarcomas, or it may be attributable to an increase in clinical awareness of these sarcomas, an increase in their reporting, an increase in the likelihood of catchment of potential tumors due to mergers of facilities, an increase in the quality of diagnostic procedures or other as yet unrecognized reasons. While the numbers of soft tissue sarcomas reported to the NCDB has increased by 19% over this 13-year time period, the number of bone sarcomas reported to the NCDB has increased by only 10.7% during this same time period (unpublished NCDB data- manuscript being prepared for publication consideration). The reason(s) for this difference is unknown.

This increase in the reported number of cases was not consistent across all histologies. There have been global changes in diagnostic tendency as evidenced by the 2002 World Health Organization declassification of MFH as a normal diagnostic entity which was renamed Undifferentiated Pleomorphic Sarcoma, NOS 25. Some of the largest increases noted during this series are Fibromyxosarcoma (326% increase), Giant-Cell Sarcoma (540% increase), Liposarcoma, dedifferentiated (171% increase), and Synovial sarcoma, spindle cell (185% increase). Two notable decreases in the number of reported cases during this 13-year interval are Fibrosarcoma, NOS (29% decrease), and MFH (54% decrease). This decrease in MFH is the largest decease in numbers over this interval and is consistent with the evolution in diagnostic tendency alluded to above. Similarly, when these tumors cannot be subclassified, many pathologists have begun to diagnose these tumors more recently as Pleomorphic Sarcoma, NOS (Senior author, pers. obs.). Consistent with this theory, the number of reported cases of Sarcoma, NOS has increased by 27% over this same time interval.

A number of previous studies of these soft tissue sarcomas have lumped together many of the various histologic subtypes when calculating survivorship rates. By analyzing each histologically distinct tumor, more accurate survival data have been generated. Because of the large number of cases presented in this report, and the fact that each center reports each diagnosed case, it is less subject to bias that can be introduced by referral patterns to individual centers, a source of potential bias in previous reports. Furthermore, by not selecting out certain patients for inclusion or exclusion as has frequently been done in previously reported series, such as selecting only patients undergoing attempts at curative resection, these data are thought to be more reflective of the true overall prognosis for unselected patients following diagnosis.

There are a number of limitations to this data set. Some are inherent in any large registry such as the NCDB. These include the establishment of the histologic diagnoses by multiple pathologists in multiple institutions. Their interpretations of the histologic material of these uncommon sarcomas could vary and may introduce bias, especially considering that certain diagnostic terms may gain or lose favor over time, such as was mentioned in the prior discussion on MFH. There may be selection bias, based on the characteristics of contributing versus noncontributing sites, but since the NCDB captures an estimated 70% of all cancers treated, it is likely that any selection bias effect is minimal due to this high level of inclusion. Another major limitation is the methodology of the case entries by hospital-based coding personnel, who largely rely on medical records which may at times be incomplete, inaccurate or improperly reported. Although the data are highly specific and well defined by the FORDS manual, some items may not be as well documented as other items. These data have been collected over a long period of time. By necessity, the documentation for and the coding of the data have involved thousands of surgeons, hospital personnel, and coding staff at hundreds of institutions. Many of those involved at any given institution have likely changed over time, adding to the numbers and potential variations in data documentation and coding.

The treatment centers may not be aware of some patient's death, as deaths may occur at facilities far removed from the original reporting treatment centers. Precise cause of death information is not reported from hospital cancer registry sources, therefore limiting computation of adjusted survival rates. However, for the various hospitals to maintain their accreditation as an ACOS CoC-accredited hospital, there is significant leverage placed on the individual institutional registries to accurately report the data. Each accredited institution must follow 90% of living patients first seen at the facility for 5 years, and 80% for the lifetime of the facility's registry, thereby ensuring a high degree of follow-up reliability http://www.facs.org/cancer/coc/programstandards2012.pdf.

In addition, the reported survivorship between each histologic subtype was not stratified based upon risk factors or treatments.

As this *β*PUF program was limited to the reporting of patients greater than 18-years old, we did not have demographic data generated for those patients under the age of 18 who have been diagnosed with soft tissue sarcomas. This presumably affected the rhabdomyosarcoma demographic data in that rhabdomyosarcomas typically predominate in children. Inclusion of the pediatric tumor data in future NCDB analyses will provide accurate epidemiologic and survival data for pediatric patients diagnosed with soft tissue sarcomas.

In summary, the data reported herein contains demographic and survivorship data on 34 distinct soft tissue sarcomas in 63,714 patients. These data are unprecedented in its scope and size and provides an accurate oversight picture of the number of such cases in the USA and the true overall associated survivorship for these entities over the years reported. These data should form a reference baseline for comparisons of future series.

## Supporting Information

**Table S1.** Anatomic distribution of 34 soft tissue tumors – Part 1. **Table S2.** Anatomic distribution of 34 soft tissue tumors – Part 2. **Table S3.** Anatomic distribution of 34 soft tissue tumors – Part 3. **Table S4.** Year of diagnosis of 34 soft tissue tumors. **Table S5.** Histologic grade and size of 34 soft tissue tumors.
