# Supplementary material for: Epidemiology and survivorship of soft tissue sarcomas in adults: a national cancer database report
Source: Cancer Med. 2014 Jul 8;3(5):1404–15. doi: 10.1002/cam4.288 (PMC4302691; doi:10.1002/cam4.288)
Supplement: Supplementary file 1 [file cam40003-1404-SD1.docx]

| **Supplemental Table 1** | | | | | | | | |
| --- | --- | --- | --- | --- | --- | --- | --- | --- |
| **Histology** | **Heart** | **Peripheral nerves and autonomic nervous system of head, face, and neck** | **Peripheral nerves and autonomic nervous system of upper limb and shoulder** | **Peripheral nerves and autonomic nervous system of lower limb and hip** | **Peripheral nerves and autonomic nervous system of thorax** | **Peripheral nerves and autonomic nervous system of abdomen** | **Peripheral nerves and autonomic nervous system of pelvis** | **Peripheral nerves and autonomic nervous system of trunk, NOS** |
| **Alveolar soft part sarcoma** | 0 | 0 | 0 | 0 | 0 | 0 | 0 | 0 |
| **Angiomyosarcoma** | 2 | 0 | 0 | 0 | 0 | 0 | 0 | 0 |
| **Chondrosarcoma, Dedifferentiated** | 0 | 0 | 0 | 0 | 0 | 0 | 0 | 0 |
| **Chondrosarcoma, Mesenchymal** | 0 | 0 | 0 | 0 | 0 | 0 | 0 | 0 |
| **Chondrosarcoma, Myxoid** | 0 | 0 | 0 | 0 | 0 | 0 | 0 | 0 |
| **Chondrosarcoma, NOS** | 1 | 2 | 1 | 0 | 0 | 0 | 0 | 0 |
| **Dermatofibrosarcoma, NOS** | 0 | 0 | 2 | 0 | 0 | 0 | 1 | 1 |
| **Ewings (including PNET)** | 1 | 4 | 7 | 16 | 7 | 5 | 11 | 12 |
| **Fibromyxosarcoma** | 10 | 0 | 3 | 1 | 0 | 0 | 0 | 0 |
| **Fibrosarcoma, NOS** | 8 | 1 | 4 | 1 | 0 | 0 | 2 | 2 |
| **Fibrous histiocytoma, malignant** | 27 | 5 | 7 | 11 | 0 | 1 | 3 | 3 |
| **Giant cell sarcoma** | 14 | 0 | 1 | 3 | 1 | 0 | 1 | 0 |
| **Hemangioendothelioma, Epithelioid, malignant** | 1 | 0 | 1 | 0 | 0 | 0 | 0 | 0 |
| **Hemangioendothelioma, malignant** | 2 | 0 | 0 | 0 | 0 | 0 | 0 | 0 |
| **Hemangiopericytoma, malignant** | 0 | 2 | 0 | 1 | 0 | 0 | 0 | 0 |
| **Hemangiosarcoma** | 266 | 8 | 2 | 2 | 0 | 2 | 0 | 1 |
| **Liposarcoma, Dedifferentiated** | 0 | 0 | 0 | 0 | 0 | 3 | 0 | 1 |
| **Liposarcoma, Myxoid** | 3 | 1 | 0 | 4 | 2 | 2 | 0 | 1 |
| **Liposarcoma, NOS** | 2 | 2 | 1 | 2 | 0 | 0 | 3 | 0 |
| **Liposarcoma, Pleomorphic** | 0 | 0 | 0 | 2 | 1 | 0 | 0 | 0 |
| **Liposarcoma, Round cell** | 1 | 0 | 0 | 0 | 0 | 0 | 0 | 0 |
| **Liposarcoma, well differentiated** | 1 | 0 | 2 | 2 | 0 | 0 | 3 | 0 |
| **Myxosarcoma** | 9 | 0 | 0 | 0 | 1 | 0 | 0 | 0 |
| **Osteosarcoma, NOS** | 12 | 0 | 0 | 0 | 0 | 0 | 0 | 0 |
| **Rhabdomyosarcoma, Alveolar** | 0 | 0 | 0 | 0 | 0 | 0 | 0 | 0 |
| **Rhabdomyosarcoma, Embryonal** | 2 | 0 | 0 | 0 | 0 | 0 | 0 | 0 |
| **Rhabdomyosarcoma, NOS** | 16 | 3 | 0 | 0 | 2 | 1 | 0 | 0 |
| **Rhabdomyosarcoma, Pleomorphic, adult type** | 3 | 0 | 1 | 0 | 0 | 0 | 1 | 0 |
| **Sarcoma, Epithelioid** | 1 | 0 | 6 | 5 | 3 | 0 | 2 | 0 |
| **Sarcoma, NOS** | 90 | 20 | 10 | 25 | 19 | 14 | 9 | 6 |
| **Sarcoma, Spindle cell** | 35 | 7 | 15 | 26 | 12 | 5 | 6 | 5 |
| **Synovial sarcoma, biphasic** | 5 | 2 | 1 | 1 | 0 | 0 | 0 | 0 |
| **Synovial sarcoma, NOS** | 15 | 2 | 11 | 6 | 1 | 0 | 0 | 0 |
| **Synovial sarcoma, spindle cell** | 8 | 0 | 5 | 2 | 3 | 1 | 1 | 0 |
| **Totals** | 535 | 59 | 80 | 110 | 52 | 34 | 43 | 32 |

| **Supplemental Table 2** | | | | | | |
| --- | --- | --- | --- | --- | --- | --- |
| **Histology** | **Overlapping lesion of peripheral nerves and autonomic nervous system** | **Autonomic nervous system, NOS** | **Connective, subcutaneous and other soft tissues of head, face, and neck** | **Connective, subcutaneous and other soft tissues of upper limb and shoulder** | **Connective, subcutaneous and other soft tissues of lower limb and hip** | **Connective, subcutaneous and other soft tissues of thorax** |
| **Alveolar soft part sarcoma** | 0 | 0 | 10 | 34 | 164 | 21 |
| **Angiomyosarcoma** | 0 | 0 | 14 | 4 | 11 | 8 |
| **Chondrosarcoma, Dedifferentiated** | 0 | 0 | 2 | 2 | 22 | 15 |
| **Chondrosarcoma, Mesenchymal** | 0 | 0 | 17 | 14 | 51 | 9 |
| **Chondrosarcoma, Myxoid** | 0 | 0 | 16 | 81 | 449 | 49 |
| **Chondrosarcoma, NOS** | 0 | 0 | 57 | 53 | 96 | 184 |
| **Dermatofibrosarcoma, NOS** | 0 | 0 | 224 | 522 | 481 | 264 |
| **Ewings (including PNET)** | 1 | 16 | 96 | 140 | 318 | 238 |
| **Fibromyxosarcoma** | 0 | 1 | 139 | 624 | 1314 | 207 |
| **Fibrosarcoma, NOS** | 0 | 1 | 176 | 347 | 622 | 262 |
| **Fibrous histiocytoma, malignant** | 0 | 2 | 1057 | 2367 | 5916 | 1030 |
| **Giant cell sarcoma** | 0 | 0 | 169 | 521 | 1591 | 292 |
| **Hemangioendothelioma, Epithelioid, malignant** | 0 | 0 | 18 | 29 | 53 | 35 |
| **Hemangioendothelioma, malignant** | 0 | 0 | 5 | 6 | 21 | 10 |
| **Hemangiopericytoma, malignant** | 0 | 1 | 79 | 33 | 157 | 40 |
| **Hemangiosarcoma** | 0 | 1 | 953 | 185 | 452 | 591 |
| **Liposarcoma, Dedifferentiated** | 1 | 0 | 26 | 110 | 406 | 102 |
| **Liposarcoma, Myxoid** | 0 | 1 | 78 | 261 | 2626 | 137 |
| **Liposarcoma, NOS** | 0 | 0 | 81 | 237 | 1143 | 148 |
| **Liposarcoma, Pleomorphic** | 0 | 0 | 43 | 285 | 684 | 133 |
| **Liposarcoma, Round cell** | 0 | 0 | 3 | 30 | 276 | 18 |
| **Liposarcoma, well differentiated** | 0 | 0 | 126 | 295 | 1737 | 220 |
| **Myxosarcoma** | 0 | 0 | 15 | 102 | 214 | 30 |
| **Osteosarcoma, NOS** | 0 | 0 | 20 | 47 | 205 | 60 |
| **Rhabdomyosarcoma, Alveolar** | 0 | 1 | 107 | 60 | 43 | 20 |
| **Rhabdomyosarcoma, Embryonal** | 0 | 1 | 64 | 15 | 39 | 18 |
| **Rhabdomyosarcoma, NOS** | 0 | 0 | 73 | 41 | 105 | 56 |
| **Rhabdomyosarcoma, Pleomorphic, adult type** | 0 | 0 | 30 | 60 | 223 | 53 |
| **Sarcoma, Epithelioid** | 0 | 1 | 42 | 292 | 283 | 74 |
| **Sarcoma, NOS** | 0 | 16 | 492 | 891 | 2390 | 880 |
| **Sarcoma, Spindle cell** | 0 | 12 | 302 | 469 | 1188 | 521 |
| **Synovial sarcoma, biphasic** | 0 | 1 | 25 | 127 | 398 | 47 |
| **Synovial sarcoma, NOS** | 0 | 5 | 87 | 320 | 893 | 162 |
| **Synovial sarcoma, spindle cell** | 0 | 0 | 95 | 210 | 516 | 138 |
| **Total** | 2 | 60 | 4741 | 8814 | 25087 | 6072 |

| **Supplemental Table 3** | | | | | | |
| --- | --- | --- | --- | --- | --- | --- |
| **Histology** | **Connective, subcutaneous and other soft tissues of abdomen** | **Connective, subcutaneous and other soft tissues of pelvis** | **Connective, subcutaneous and other soft tissues of trunk, NOS** | **Overlapping lesion of connective, subcutaneous and other soft tissues** | **Connective, subcutaneous, and other soft tissues, NOS** | **Total** |
| **Alveolar soft part sarcoma** | 15 | 35 | 7 | 0 | 20 | 306 |
| **Angiomyosarcoma** | 6 | 5 | 2 | 0 | 3 | 55 |
| **Chondrosarcoma, Dedifferentiated** | 0 | 7 | 3 | 1 | 0 | 52 |
| **Chondrosarcoma, Mesenchymal** | 6 | 20 | 3 | 0 | 5 | 125 |
| **Chondrosarcoma, Myxoid** | 21 | 98 | 20 | 5 | 15 | 754 |
| **Chondrosarcoma, NOS** | 16 | 69 | 27 | 1 | 20 | 527 |
| **Dermatofibrosarcoma, NOS** | 273 | 214 | 388 | 10 | 37 | 2417 |
| **Ewings (including PNET)** | 121 | 214 | 78 | 9 | 46 | 1340 |
| **Fibromyxosarcoma** | 101 | 223 | 165 | 7 | 40 | 2835 |
| **Fibrosarcoma, NOS** | 171 | 186 | 142 | 7 | 45 | 1977 |
| **Fibrous histiocytoma, malignant** | 478 | 893 | 719 | 39 | 196 | 12754 |
| **Giant cell sarcoma** | 183 | 342 | 197 | 15 | 85 | 3415 |
| **Hemangioendothelioma, Epithelioid, malignant** | 10 | 8 | 6 | 1 | 29 | 191 |
| **Hemangioendothelioma, malignant** | 2 | 5 | 1 | 0 | 14 | 66 |
| **Hemangiopericytoma, malignant** | 53 | 87 | 32 | 2 | 42 | 529 |
| **Hemangiosarcoma** | 203 | 136 | 139 | 17 | 414 | 3372 |
| **Liposarcoma, Dedifferentiated** | 404 | 292 | 85 | 19 | 60 | 1509 |
| **Liposarcoma, Myxoid** | 234 | 416 | 164 | 16 | 50 | 3996 |
| **Liposarcoma, NOS** | 406 | 338 | 153 | 25 | 83 | 2624 |
| **Liposarcoma, Pleomorphic** | 137 | 131 | 106 | 6 | 23 | 1551 |
| **Liposarcoma, Round cell** | 22 | 33 | 20 | 3 | 4 | 410 |
| **Liposarcoma, well differentiated** | 453 | 620 | 197 | 48 | 90 | 3794 |
| **Myxosarcoma** | 22 | 46 | 31 | 3 | 11 | 484 |
| **Osteosarcoma, NOS** | 20 | 40 | 16 | 3 | 15 | 438 |
| **Rhabdomyosarcoma, Alveolar** | 15 | 56 | 8 | 9 | 29 | 348 |
| **Rhabdomyosarcoma, Embryonal** | 16 | 64 | 9 | 2 | 24 | 254 |
| **Rhabdomyosarcoma, NOS** | 56 | 87 | 10 | 8 | 47 | 505 |
| **Rhabdomyosarcoma, Pleomorphic, adult type** | 52 | 61 | 24 | 4 | 14 | 526 |
| **Sarcoma, Epithelioid** | 57 | 177 | 45 | 5 | 42 | 1035 |
| **Sarcoma, NOS** | 939 | 1085 | 380 | 59 | 517 | 7842 |
| **Sarcoma, Spindle cell** | 435 | 491 | 186 | 30 | 182 | 3927 |
| **Synovial sarcoma, biphasic** | 40 | 45 | 17 | 4 | 19 | 732 |
| **Synovial sarcoma, NOS** | 71 | 119 | 56 | 9 | 63 | 1820 |
| **Synovial sarcoma, spindle cell** | 66 | 80 | 28 | 9 | 42 | 1204 |
| **Total** | 5104 | 6723 | 3464 | 376 | 2326 | 63714 |

| **Supplemental Table 4** | | | | | | | | | | | | | | |
| --- | --- | --- | --- | --- | --- | --- | --- | --- | --- | --- | --- | --- | --- | --- |
|  | **Year of Diagnosis** | | | | | | | | | | | | | |
| **Histology** | **1998** | **1999** | **2000** | **2001** | **2002** | **2003** | **2004** | **2005** | **2006** | **2007** | **2008** | **2009** | **2010** | **Total** |
| **Alveolar soft part sarcoma** | 16 | 22 | 23 | 19 | 23 | 26 | 22 | 24 | 22 | 21 | 38 | 35 | 15 | 306 |
| **Angiomyosarcoma** | 2 | 8 | 10 | 6 | 4 | 3 | 2 | 3 | 1 | 6 | 5 | 3 | 2 | 55 |
| **Chondrosarcoma, Dedifferentiated** | 0 | 0 | 0 | 2 | 3 | 3 | 7 | 4 | 7 | 3 | 12 | 6 | 5 | 52 |
| **Chondrosarcoma, Mesenchymal** | 10 | 5 | 4 | 9 | 12 | 10 | 16 | 9 | 12 | 12 | 8 | 9 | 9 | 125 |
| **Chondrosarcoma, Myxoid** | 54 | 43 | 40 | 42 | 63 | 34 | 51 | 63 | 56 | 75 | 69 | 90 | 74 | 754 |
| **Chondrosarcoma, NOS** | 47 | 36 | 35 | 32 | 23 | 42 | 49 | 40 | 34 | 51 | 46 | 54 | 38 | 527 |
| **Dermatofibrosarcoma, NOS** | 189 | 181 | 192 | 221 | 207 | 185 | 167 | 184 | 154 | 178 | 160 | 155 | 244 | 2417 |
| **Ewings (including PNET)** | 80 | 76 | 96 | 116 | 79 | 100 | 118 | 121 | 112 | 117 | 126 | 107 | 92 | 1340 |
| **Fibromyxosarcoma** | 75 | 92 | 107 | 110 | 143 | 163 | 183 | 230 | 265 | 303 | 353 | 367 | 444 | 2835 |
| **Fibrosarcoma, NOS** | 168 | 173 | 162 | 156 | 184 | 173 | 185 | 135 | 163 | 126 | 117 | 123 | 112 | 1977 |
| **Fibrous histiocytoma, malignant** | 1333 | 1220 | 1281 | 1174 | 1195 | 1110 | 1096 | 1006 | 868 | 736 | 633 | 584 | 518 | 12754 |
| **Giant cell sarcoma** | 73 | 79 | 89 | 121 | 122 | 153 | 193 | 266 | 344 | 440 | 465 | 529 | 541 | 3415 |
| **Hemangioendothelioma, Epithelioid, malignant** | 12 | 18 | 9 | 13 | 19 | 7 | 15 | 14 | 18 | 18 | 11 | 24 | 13 | 191 |
| **Hemangioendothelioma, malignant** | 12 | 2 | 5 | 7 | 4 | 10 | 2 | 6 | 5 | 5 | 3 | 3 | 2 | 66 |
| **Hemangiopericytoma, malignant** | 51 | 46 | 49 | 49 | 36 | 37 | 31 | 47 | 39 | 37 | 47 | 26 | 34 | 529 |
| **Hemangiosarcoma** | 193 | 213 | 239 | 237 | 249 | 211 | 249 | 270 | 282 | 304 | 274 | 322 | 329 | 3372 |
| **Liposarcoma, Dedifferentiated** | 62 | 67 | 69 | 100 | 72 | 91 | 113 | 118 | 132 | 149 | 170 | 186 | 180 | 1509 |
| **Liposarcoma, Myxoid** | 279 | 281 | 289 | 317 | 341 | 305 | 270 | 308 | 305 | 326 | 335 | 337 | 303 | 3996 |
| **Liposarcoma, NOS** | 210 | 184 | 215 | 179 | 201 | 206 | 218 | 184 | 206 | 227 | 223 | 194 | 177 | 2624 |
| **Liposarcoma, Pleomorphic** | 121 | 121 | 123 | 115 | 106 | 110 | 102 | 128 | 115 | 128 | 120 | 131 | 131 | 1551 |
| **Liposarcoma, Round cell** | 27 | 29 | 29 | 33 | 29 | 29 | 33 | 44 | 30 | 32 | 32 | 40 | 23 | 410 |
| **Liposarcoma, well differentiated** | 193 | 191 | 239 | 253 | 256 | 275 | 296 | 315 | 274 | 350 | 335 | 413 | 404 | 3794 |
| **Myxosarcoma** | 19 | 27 | 17 | 36 | 19 | 32 | 25 | 40 | 54 | 62 | 51 | 49 | 53 | 484 |
| **Osteosarcoma, NOS** | 25 | 29 | 27 | 17 | 23 | 32 | 46 | 41 | 41 | 35 | 45 | 32 | 45 | 438 |
| **Rhabdomyosarcoma, Alveolar** | 20 | 22 | 24 | 24 | 27 | 24 | 31 | 25 | 18 | 27 | 38 | 39 | 29 | 348 |
| **Rhabdomyosarcoma, Embryonal** | 10 | 21 | 24 | 12 | 18 | 13 | 25 | 21 | 20 | 21 | 19 | 29 | 21 | 254 |
| **Rhabdomyosarcoma, NOS** | 27 | 33 | 40 | 33 | 43 | 33 | 35 | 41 | 32 | 53 | 44 | 49 | 42 | 505 |
| **Rhabdomyosarcoma, Pleomorphic, adult type** | 22 | 28 | 25 | 30 | 34 | 55 | 40 | 36 | 34 | 53 | 52 | 55 | 62 | 526 |
| **Sarcoma, Epithelioid** | 54 | 68 | 76 | 67 | 66 | 67 | 86 | 75 | 84 | 79 | 114 | 104 | 95 | 1035 |
| **Sarcoma, NOS** | 520 | 554 | 563 | 469 | 525 | 575 | 613 | 636 | 648 | 653 | 710 | 693 | 683 | 7842 |
| **Sarcoma, Spindle cell** | 271 | 262 | 285 | 277 | 273 | 307 | 262 | 330 | 334 | 314 | 325 | 360 | 327 | 3927 |
| **Synovial sarcoma, biphasic** | 57 | 47 | 55 | 44 | 63 | 63 | 53 | 60 | 60 | 57 | 56 | 46 | 71 | 732 |
| **Synovial sarcoma, NOS** | 160 | 173 | 166 | 120 | 144 | 145 | 132 | 144 | 117 | 124 | 131 | 134 | 130 | 1820 |
| **Synovial sarcoma, spindle cell** | 35 | 42 | 48 | 98 | 102 | 93 | 107 | 98 | 122 | 115 | 111 | 111 | 122 | 1204 |
| **Total** | 4427 | 4393 | 4655 | 4538 | 4708 | 4722 | 4873 | 5066 | 5008 | 5237 | 5278 | 5439 | 5370 | 63714 |

| **Supplemental Table 5** | | | | | | | |
| --- | --- | --- | --- | --- | --- | --- | --- |
| **Histology** | **Well differentiated, differentiated, NOS** | **Moderately differentiated, moderately well differentiated, intermediate differentiation** | **Poorly differentiated** | **Undifferentiated, anaplastic** | **Cell type not determined, not stated or not applicable, unknown primaries, high grade dysplasia** | **Size 5+cm** | **Size <5cm** |
| **Alveolar soft part sarcoma** | 8 | 13 | 35 | 23 | 227 | 187 | 57 |
| **Angiomyosarcoma** | 5 | 2 | 17 | 5 | 26 | 22 | 9 |
| **Chondrosarcoma, Dedifferentiated** | 3 | 2 | 15 | 24 | 8 | 43 | 5 |
| **Chondrosarcoma, Mesenchymal** | 3 | 6 | 31 | 28 | 57 | 81 | 22 |
| **Chondrosarcoma, Myxoid** | 159 | 170 | 97 | 47 | 281 | 467 | 177 |
| **Chondrosarcoma, NOS** | 191 | 160 | 64 | 25 | 87 | 302 | 113 |
| **Dermatofibrosarcoma, NOS** | 251 | 120 | 65 | 20 | 1961 | 578 | 1152 |
| **Ewings (including PNET)** | 13 | 11 | 212 | 261 | 843 | 720 | 246 |
| **Fibromyxosarcoma** | 744 | 727 | 635 | 436 | 293 | 1488 | 1016 |
| **Fibrosarcoma, NOS** | 398 | 482 | 476 | 250 | 371 | 971 | 589 |
| **Fibrous histiocytoma, malignant** | 415 | 1200 | 4473 | 3147 | 3519 | 6711 | 3792 |
| **Giant cell sarcoma** | 36 | 137 | 1262 | 1538 | 442 | 2164 | 785 |
| **Hemangioendothelioma, Epithelioid, malignant** | 30 | 29 | 13 | 4 | 115 | 31 | 94 |
| **Hemangioendothelioma, malignant** | 10 | 12 | 3 | 5 | 36 | 10 | 25 |
| **Hemangiopericytoma, malignant** | 82 | 73 | 56 | 36 | 282 | 318 | 114 |
| **Hemangiosarcoma** | 223 | 256 | 984 | 518 | 1391 | 1049 | 1068 |
| **Liposarcoma, Dedifferentiated** | 153 | 108 | 472 | 519 | 257 | 1096 | 232 |
| **Liposarcoma, Myxoid** | 1273 | 933 | 458 | 217 | 1115 | 2893 | 609 |
| **Liposarcoma, NOS** | 1414 | 211 | 321 | 182 | 496 | 1794 | 338 |
| **Liposarcoma, Round cell** | 24 | 43 | 180 | 72 | 91 | 315 | 53 |
| **Liposarcoma, Pleomorphic** | 50 | 70 | 669 | 445 | 317 | 1075 | 303 |
| **Liposarcoma, well differentiated** | 3614 | 55 | 25 | 16 | 84 | 2828 | 433 |
| **Myxosarcoma** | 94 | 95 | 107 | 82 | 106 | 233 | 166 |
| **Osteosarcoma, NOS** | 10 | 18 | 154 | 157 | 99 | 255 | 108 |
| **Rhabdomyosarcoma, Alveolar** | 3 | 3 | 75 | 44 | 223 | 130 | 63 |
| **Rhabdomyosarcoma, Embryonal** | 5 | 6 | 60 | 44 | 139 | 122 | 53 |
| **Rhabdomyosarcoma, NOS** | 6 | 7 | 149 | 82 | 261 | 285 | 62 |
| **Rhabdomyosarcoma, Pleomorphic, adult type** | 1 | 6 | 217 | 160 | 142 | 350 | 85 |
| **Sarcoma, Epithelioid** | 36 | 59 | 292 | 188 | 460 | 387 | 417 |
| **Sarcoma, NOS** | 649 | 601 | 2416 | 1739 | 2437 | 3991 | 1489 |
| **Sarcoma, Spindle cell** | 317 | 516 | 1114 | 857 | 1123 | 2159 | 817 |
| **Synovial sarcoma, biphasic** | 32 | 106 | 210 | 85 | 299 | 372 | 245 |
| **Synovial sarcoma, NOS** | 56 | 154 | 605 | 253 | 752 | 955 | 469 |
| **Synovial sarcoma, spindle cell** | 38 | 143 | 366 | 178 | 479 | 636 | 354 |
| **Total** | 10346 | 6534 | 16328 | 11687 | 18819 | 35018 | 15560 |
